# Post-Surgical Ulnar Nerve Neuropathy in Distal Humerus Fractures: Comparison Between In Situ Decompression and Anterior Subcutaneous Transposition

**DOI:** 10.3390/jcm14072490

**Published:** 2025-04-05

**Authors:** Ignacio García-Cepeda, Ana-Elena Sanz-Peñas, Inés de Blas-Sanz, Clarisa Simón-Pérez, Emilio-Javier Frutos-Reoyo, Ignacio Aguado-Maestro

**Affiliations:** 1 Department of Traumatology and Orthopaedic Surgery, Hospital Universitario del Río Hortega, C Dulzaina 2, 47012 Valladolid, Spain; icepeda@saludcastillayleon.es (I.G.-C.); asanzpe@saludcastillayleon.es (A.-E.S.-P.); 2 Department of Traumatology and Orthopaedic Surgery, Complejo Asistencial de Palencia, 34005 Palencia, Spain; ideblassanzd@saludcastillayleon.es; 3 Department of Traumatology and Orthopaedic Surgery, Hospital Clínico Universitario, 47003 Valladolid, Spain; csimon@saludcastillayleon.es; 4 Department of Rehabilitation and Physical Medicine, Hospital Universitario del Río Hortega, C Dulzaina 2, 47012 Valladolid, Spain; efrutosr@saludcastillayleon.es

**Keywords:** humerus, fractures, ulnar neuropathy, nerve transposition, ulnar nerve

## Abstract

Postoperative ulnar neuropathy is a common complication in patients undergoing surgery for distal humerus fractures, particularly when open reduction and internal fixation (ORIF) is performed. **Objectives**: This study aims to compare the rates of ulnar nerve neuropathy following classic in situ decompression versus ulnar nerve subcutaneous anterior transposition. **Methods**: A retrospective study was conducted, including 51 patients treated for distal humerus fractures with precontoured locking plates in our institution between 2009 and 2023, according to specific inclusion and exclusion criteria. Age, sex, ulnar neuropathy, range of motion (ROM), complications, surgical approach, and Mayo Elbow Performance Score (MEPS) were evaluated. Ulnar nerve function was graded according to modified McGowan classification. **Results**: Ulnar neuropathy was observed in 17 (33.3%) patients, with a higher risk in those who underwent anterior ulnar nerve transposition compared to in situ decompression (58% vs. 26%, *p* = 0.042). In the modified McGowan classification, 14 patients had grade 1 and 3 had grade 2 neuropathy. The overall complication rate was 49%, and the functional outcomes according to the MEPS scale showed a mean score of 81.6 (SD 17.29). The mean flexion–extension was 100.56°, and 94% of patients retained complete pronosupination. **Conclusions**: Our results demonstrate that routine intraoperative ulnar nerve transposition should not be performed for these fractures.

## 1. Introduction

Distal humerus fractures (DHFs) constitute a small percentage of all fractures, with an incidence of 2–6% and 30% of elbow fractures [1,2]. Although uncommon, they present significant complexity due to their potential involvement of the articular surface of the humerus, complicating both surgical repair and the overall functional prognosis of the joint [3,4]. These fractures exhibit a bimodal age distribution, occurring in young men, who often experience high-energy trauma, and elderly women, whose fractures are generally the result of low-energy falls associated with postmenopausal osteoporosis [2,3,5].

Surgical management is generally required, with open reduction and internal fixation (ORIF) using precontoured locking plates being the standard treatment [6]. Various surgical approaches are available: the paratricipital is recommended for extra-articular or uncomplicated intra-articular fractures [7,8,9], while olecranon osteotomy provides improved access to intra-articular DHFs [10,11]. In elderly patients, comminution and fragment deformation can be severe; therefore, elbow arthroplasty may be considered in the treatment of these patients [5,12]. The surgical management required for the treatment of these fractures through ORIF also leads to an increase in inflammatory markers in the blood, which is more pronounced compared to what occurs in less invasive surgeries [13]. This increased inflammatory response has been correlated with poorer survival outcomes following major surgeries [14].

Common complications of DHFs include ulnar neuropathy, hardware intolerance, and limited range of motion [5,15]. In addition to these complications, surgical trauma to the ulnar nerve during ORIF is a significant concern. There is substantial evidence supporting subcutaneous anterior transposition of the ulnar nerve in patients with preoperative signs of ulnar neuropathy [16,17]. However, the optimal approach to ulnar nerve management during ORIF for distal humerus fractures remains debated in patients without preoperative neurological deficits. The ulnar nerve, which runs near the medial side of the elbow, is frequently exposed during ORIF, making it vulnerable to injury, but its intraoperative handling varies among surgeons. While some prefer in situ decompression [16,18], others routinely perform nerve transposition [11,19,20,21]. Some studies have shown that surgical trauma, including stretching or compressing the nerve, can result in damage to the nerve’s blood supply, leading to an increased risk of neuropathy following surgery [16,17]. Additionally, other authors suggest that anterior transposition may compromise the nerve’s blood supply [22] and therefore do not recommend routine transposition during DHF surgery [23].

The primary aim of this study is to compare the incidence of postoperative ulnar neuropathy in patients undergoing ulnar nerve transposition versus in situ decompression during ORIF for DHFs. Secondary objectives include analyzing the overall complications and evaluating functional outcomes post-surgery.

## 2. Materials and Methods

### 2.1. Study Design

This observational and retrospective study was conducted in the Trauma and Orthopedic Surgery Department of Río Hortega University Hospital. A total of four experienced surgeons with more than 10 years of practice and fellowship training in traumatology participated in the surgeries. A power analysis was not performed due to the retrospective nature of the study.

### 2.2. Eligibility Criteria

We included all patients aged 18 years and older who underwent ORIF with precontoured locking plates for isolated DHFs between January 2009 and October 2023 with at least 6 months of follow-up.

Patients younger than 18 years, those with preoperative ulnar nerve neuropathy or concomitant injuries (e.g., ipsilateral distal radius fractures, bilateral distal humerus fractures, polytrauma), and those treated with other implants such as nonlocking plates, arthroplasty, or isolated screws were excluded.

### 2.3. Data Collection

Medical records were reviewed retrospectively. The following information was collected for each patient: patient sex and age, mechanism of injury, operation and tourniquet time, intraoperative ulnar nerve management, and common complications like surgical site infection (superficial or deep), heterotopic ossification, ulnar neuropathy, fracture consolidation, and the need for reoperation. Range of motion (ROM) was measured using a long-arm goniometer, and subjective assessment was performed using the Mayo Elbow Performance Score (MEPS). The MEPS survey, conducted 6 months post-surgery, evaluates pain, joint mobility, stability, and daily activity performance [24].

### 2.4. Surgical Technique

Patients were positioned prone, with the arm flexed at 90 and supported on an armrest. The intervention began with a posterior approach to the elbow, and the ulnar nerve was released at the cubital tunnel, followed by meticulous dissection along the medial aspect of the triceps, extending proximally. Depending on the surgeon’s judgment and the fracture complexity, a triceps-sparing technique with medial and lateral windows or an olecranon osteotomy was performed. Fixation was accomplished using precontoured locking plates (Acumed Elbow Plating System, Hillsboro, OR, USA or Variable Angle LCP Elbow System DePuy Synthes, Raynham, MA, USA) with different configurations, orthogonal (90°) or parallel (180°), which were chosen based on intraoperative assessment.

The decision to perform either decompression or anterior transposition of the ulnar nerve was made at the discretion of the surgeon during the surgery. This indication was based on intraoperative findings and the surgeon’s judgment. Specifically, the nerve was assessed for any contact with the fixation plate or if it appeared unstable upon its repositioning in the cubital tunnel. If the nerve was found to be in contact with the plate or at risk of becoming unstable, the surgeon usually opted for anterior transposition. If the nerve could be safely repositioned without tension and without direct contact with the hardware, in situ decompression was performed. This decision-making process was entirely based on the surgeon’s intraoperative assessment and experience.

When anterior transposition was performed, the ulnar nerve was fully mobilized and released distally between the two heads of the flexor carpi ulnaris muscle. The nerve was then carefully visualized and tagged with vessel loops throughout the procedure. It was secured in the subcutaneous tissue by placing it under the fascia to ensure its proper position and prevent any displacement. In the non-transposition group, the ulnar nerve was repositioned behind the medial epicondyle.

At the end of the surgery, olecranon osteosynthesis (if performed) was completed using a cannulated 6.5 mm cancellous screw (DePuy Synthes, Raynham, MA, USA) or K-wires with a tension band depending on the surgeon’s preference.

### 2.5. Evaluation of Ulnar Nerve Injury

The presence and severity of neuropathy were evaluated both directly after surgery and during the final follow-up visit. Neurological examination assessed motor and sensory function of the elbow nerves using the modified McGowan [25] classification to evaluate the severity of neuropathy. Grade 1 was characterized by minimal lesions with no detectable motor dysfunction in the ulnar intrinsic muscles, marked by paresthesia in the ulnar nerve distribution and only mild sensory loss. Grade 2 reflected more severe lesions, with reduced strength of the interossei muscles and muscle degeneration, accompanied by marked sensory impairment. Grade 3 represented a severe lesion, with complete paralysis of the interossei muscles and significant hypoesthesia.

### 2.6. Radiographic Evaluation

Preoperative radiographic and Computed Tomography images were evaluated and classified according to the AO/OTA fracture classification system [26]. Fractures were categorized into three types: extra-articular (AO/OTA type A), partial articular (AO/OTA type B), and intra-articular (AO/OTA type C). Follow-up assessments included anteroposterior and lateral radiographs to evaluate fracture healing, the development of post-traumatic osteoarthritis, implant failure, and heterotopic ossification.

### 2.7. Statistical Analysis

Statistical analysis was performed to evaluate the association between postoperative ulnar neuropathy and intraoperative nerve transposition, as well as the influence of different plate configurations and other clinical variables. Quantitative variables were described as mean ± standard deviation (SD), and categorical variables as absolute frequencies and percentages. The normality of continuous variables was assessed using the Shapiro–Wilk test, which is recommended for small sample sizes. Depending on the results, comparisons between groups were conducted using Student’s t-test for normally distributed variables or the Mann–Whitney U test when the distribution was non-normal. Categorical variables were analyzed using the chi-square test or Fisher’s exact test, as appropriate. All statistical analyses were performed using SPSS Statistics for Mac (version 29, IBM, Armonk, NY, USA), and a *p*-value < 0.05 was considered statistically significant.

## 3. Results

A total of 51 patients sustained distal humerus fractures during the designated study period. Among them, 25 were women (49%) and 26 were men (51%). The mean age at the time of surgery was 56 years (standard deviation [SD]: 21.6; range: 18–86), with an average follow-up period of 11.5 months (SD: 5.93, R: 6–24 months).

The fractures were categorized as 9 AO type A (7 A2 and 2 A3) and 42 AO type C (10 C1, 13 C2, and 19 C3). All patients underwent ORIF using precontoured plates, with 84% also requiring an olecranon osteotomy. Of these, 11 (26%) were fixed with a 6.5 mm partial-thread titanium cannulated screw and 32 (74%) were fixed with K-wires and a tension band. A total of 9 plates were placed perpendicular (18%), and 42 plates (82%) were placed parallel.

The overall complication rate was 49% (25/51). Ulnar neuropathy in various degrees was the most frequent complication, observed in 17 patients (33%). In addition to ulnar neuropathy, other complications were encountered that could potentially have influenced the results, including three cases of nonunion (pseudoarthrosis), four cases of surgical wound infections, and two cases of wound dehiscence. Furthermore, four patients developed elbow stiffness, and three patients experienced radial nerve palsies, all of which were temporary and resolved with no permanent deficits. In terms of reinterventions, 15 patients had intolerance to the osteosynthesis hardware requiring its removal, 1 patient with nonunion underwent a revision surgery, 1 patient required irrigation and debridement due to infection, 4 patients underwent ulnar nerve release for persistent neuropathy without clinical improvement, and 2 patients required treatment for elbow stiffness (Table 1).

Intraoperative management of the ulnar nerve consisted of in situ release in 76.5% of cases and anterior transposition in 23.5%. The overall incidence of ulnar neuropathy was 33.3%, with a higher rate in the transposition group (58%, 7/12) compared to the in situ decompression group (26%, 10/39). This difference was statistically significant (*p* = 0.042), suggesting a lower risk of neuropathy in patients who did not undergo nerve transposition. Of the 17 patients who developed ulnar neuropathy, 14 (82%) had grade 1 in the modified McGowan classification and 3 (18%) had grade 2 neuropathy. No significant association was found between the performance of olecranon osteotomy and the development of ulnar neuropathy (*p* = 0.785). However, ulnar neuropathy was significantly more frequent in patients who underwent ORIF with a 180° (parallel) plate configuration compared to those with a 90° (orthogonal) configuration (0% vs. 40.5%; *p* = 0.021) (Table 2). Among the three patients who required a secondary surgical procedure for persistent ulnar neuropathy after transposition, all were classified as McGowan grade 1. Regarding the severity of postoperative neuropathy, three patients were classified as McGowan grade 2: two of them had undergone in situ decompression and one had received an anterior transposition.

The data regarding the range of flexion–extension showed a mean of 100.56° (SD: 28, range: 20–145°). Regarding pronosupination, full range of motion was achieved in 47 patients, while 4 patients experienced some degree of limitation (10–30°). Finally, the mean Mayo Elbow Performance Score (MEPS) at 6 months post-surgery was 81.60 (SD: 17.29, range: 45–100).

## 4. Discussion

Treating fractures of the distal humerus is particularly challenging due to the nearby neurovascular structures and the complex nature of the fracture patterns. Ulnar neuropathy is a common complication following surgery for distal humerus fractures [27]. Many approaches have been suggested to minimize this risk, though the optimal method remains uncertain. Ulnar neuropathy can result from several factors, with iatrogenic causes including hardware, aggressive retraction, fracture realignment, or devascularization [27].

The most common approach for these fractures involves exposing the ulnar nerve; however, there is debate about whether it is better to perform an anterior transposition of the nerve or in situ release alone [9]. Anterior transposition offers several benefits, such as preventing the nerve from becoming twisted or deformed if not fully freed, relocating the nerve further from the hardware, preventing anterior dislocation over the medial condyle, reducing the chance of nerve entrapment in scar tissue, and decreasing its tension along its path [28]. However, several mechanisms may explain why transposition is associated with a higher incidence of postoperative ulnar neuropathy in our series. This procedure can lead to complications, including increased manipulation of the nerve (which could raise the risk of a neuroapraxic injury), fibrosis around the nerve, and disruption of its blood supply [29]. Inadequate release of surrounding tissue, such as the medial intermuscular septum, can also cause nerve compression. On the other hand, in situ release is simpler, involves less nerve handling, preserves its blood supply, and reduces the risk of traction injuries. Despite this, in this approach, the tension on the ulnar nerve may be higher because it is placed in contact with the medial hardware [30].

Several recommendations have been made regarding the management of the ulnar nerve in distal humerus fractures. Some authors choose to transpose the nerve if it is necessary to move the ulnar nerve to avoid iatrogenic damage, if the fixation hardware is placed in the ulnar groove, or if the ulnar nerve is compressed by the hardware during elbow flexion [18,31,32]. Other surgeons transpose the nerve without a clear justification or prefer to transpose it in all cases [11,19,20], while others do not transpose it but temporarily move it during surgery and then return it to the cubital tunnel at the end of the procedure [11,19].

The management of the ulnar nerve in DHF surgery in patients with preoperative neuropathy has gained increasing evidence through studies by Ruan et al. [16] that examined the effectiveness of in situ decompression versus anterior subfascial transposition for managing ulnar nerve issues in patients with preoperative symptoms. A total of 29 patients with preoperative ulnar symptoms were randomly assigned to either surgical procedure. In the transposition group, 80% of patients experienced full recovery, while 20% showed partial improvement. In the in situ group, 57% had complete recovery and 43% had partial recovery. Additionally, 86.7% of the transposition group achieved excellent or good outcomes, compared to only 57.1% in the in situ group. These results suggest that subfascial transposition offers better outcomes than in situ decompression. Later, Nauth et al. [9] conducted a systematic review and concluded that there is enough evidence (grade B) to endorse anterior ulnar nerve transposition when performing ORIF on distal humerus fractures in patients with preexisting ulnar nerve symptoms.

The surgical approach to managing the ulnar nerve in the absence of preoperative dysfunction during surgical treatment of DHF can involve either subcutaneous anterior transposition or in situ decompression. Despite this, the most effective method remains a topic of debate.

In this retrospective study, we included patients without preoperative neuropathy, and the overall rate of postoperative ulnar nerve neuropathy was 33.3%, with 58.3% and 25.6% in the transposition and no transposition groups, respectively. Performing an ulnar nerve transposition at the time of surgery was associated with a significant (*p* = 0.042) increase in the risk of developing ulnar nerve neuropathy.

Additionally, we investigated the incidence of ulnar neuropathy in fractures treated with orthogonal plates (0%) compared to parallel plates (40.5%) (*p* = 0.021). This statistically significant difference is thought to be attributable to the small number of patients (*n* = 9) treated with orthogonal plates, with only two cases involving ulnar nerve transposition. This finding was confirmed in a recent meta-analysis [33] that reported no statistically significant difference between the parallel and orthogonal plating groups in terms of ulnar neuropathy (RR = 0.52, 95% CI = 0.26 to 1.07, *p* = 0.08), which may be due to the larger sample size compared to our study.

McKee et al. [17] conducted ulnar nerve transposition on 21 patients with ulnar nerve dysfunction, achieving favorable outcomes in 17 of the cases. As a result, routine ulnar nerve transposition was considered a preventative measure to avoid ulnar nerve neuropathy after DHF. Despite this theoretical advantage, several investigations have shown that transposition does not reduce the occurrence of postoperative ulnar nerve neuropathy. For instance, Wiggers et al. [34] studied 107 patients with DHF and found that 17% developed neuropathy regardless of whether transposition was performed. Similarly, studies by Worden [30], Vazquez [28], Dehghan [35], and Oshika et al. [36] found no significant difference between transposition and in situ decompression in preventing ulnar nerve dysfunction. Conversely, some research suggests that performing ulnar nerve transposition during distal humerus surgery may increase the risk of neuropathy. Chen et al. [23] reported a fourfold increase in the incidence of ulnar nerve neuropathy in patients who underwent transposition compared to those who did not. Ahmed et al. [37] also reported that ulnar nerve transposition resulted in a 4.8 times greater risk of postoperative dysfunction. Since we found a 2.3 times greater risk, we do not recommend transposition if neuropathy is not observed prior to surgery (Table 3).

Among the patients who required reoperation despite anterior transposition, all were classified as McGowan grade 1. Additionally, two out of three patients with McGowan grade 2 neuropathy had undergone in situ decompression, while one had received a transposition. However, due to the small number of cases with severe neuropathy or revision surgery, no definitive conclusions can be drawn regarding a potential association between the severity of ulnar neuropathy and the type of nerve management. These observations should be interpreted with caution and further investigated in larger cohorts.

Interestingly, the presence of ulnar nerve neuropathy did not result in significantly lower MEPS scores in our cohort. This may be explained by the fact that the MEPS primarily evaluates pain, stability, and range of motion, and does not specifically capture neurological symptoms such as paresthesia or subtle motor deficits. Additionally, most cases of neuropathy in our study were classified as grade 1, and patients may have compensated functionally despite mild sensory changes. It is also possible that the follow-up period was not sufficient to detect long-term effects of persistent neuropathy on overall elbow function.

The elevated complication rate observed in this study (49%), as well as in previous studies (18–53%), highlights the challenges in treating DHF [12]. The most frequently reported complications include ulnar neuropathy, wound infection, elbow stiffness, implant removal, malunion, and nonunion [12,38]. The second most frequent complication we found was symptomatic implant removal of olecranon osteotomy, with an incidence of 35% (15/43), higher than in Haglin et al. [39] (6%) but lower than in Rosenlund et al. [5] (45%). It was noted that the screw fixation group had a lower incidence of removal compared to the tension band wiring group (9% vs. 25%), although this difference was not statistically significant (*p* = 0.213). Despite the high incidence of intolerance, Haglin [39] noted that some authors believe olecranon osteotomy should be prioritized due to its association with fewer postoperative complications of DHF, which have greater morbidity than intolerance to olecranon fixation material. Other authors, like Rosenlund [5], suggest considering alternative exposure methods that do not require osteotomy. We reported a secondary surgical rate of 33%, consistent with the 21–79% range found in previous studies [40].

This study has several limitations. First, as a retrospective study utilizing a clinical database, the lack of randomization introduces a potential for selection bias. Second, the short follow-up period may lead to an overestimation of ulnar nerve neuropathy cases, as some patients might improve with extended follow-up. However, Chen et al. [23] reported that six months is sufficient to detect ulnar symptoms related to the initial trauma and surgery, while the time required for neuropathy to develop due to scar tissue formation, heterotopic ossification, or prominent hardware remains uncertain. Third, neuropathy assessment was based solely on clinical evaluation, without the use of Semmes–Weinstein monofilament testing or electrodiagnostic studies. Consequently, the true incidence of neurological injury may have been underestimated.

## 5. Conclusions

The findings of this study suggest that ulnar nerve transposition during open reduction and internal fixation (ORIF) of distal humerus fractures, in the absence of preexisting neuropathy, is associated with an increased risk of developing ulnar nerve neuropathy compared to in situ decompression. As a result, routine ulnar nerve transposition during ORIF for distal humerus fractures is not supported by our findings and therefore is not recommended. Further prospective, randomized studies are warranted to clarify the potential benefits of ulnar nerve transposition in this context.

## Figures and Tables

**Table 1:** Complications and secondary surgical procedures.

Complication	Number of Cases	Reintervention Performed
Ulnar neuropathy	17	4 ulnar nerve release
Hardware intolerance	15	15 implant removal
Stiffness	4	2 elbow release
Surgical wound infection	4	1 wound washout
Radial nerve palsy	3	0 radial nerve release
Nonunion	2	1 graft + ORIF

ORIF: Open reduction and internal fixation.

**Table 2:** Comparison of groups.

Variables	Ulnar Neuropathy (*n* = 17)	No Neuropathy (*n* = 34)	*p*-Value
Sex			
Male	41.2%	55.9%	
Female	58.8%	44.1%	0.322 ^1^
Age	56.7 years	55.4 years	0.803 ^5^
Type of fracture (AO/OTA)			
A2	2 (11.8%)	5 (14.7%)	
A3	0 (0%)	2 (5.9%)	
C1	4 (23.5%)	6 (17.6%)	
C2	4 (23.5%)	9 (26.5%)	
C3	7 (41.2%)	12 (35.3%)	0.730 ^3^
Tourniquet time	124.4 min	126.7 min	0.498 ^5^
Plate configuration			
90°	0 (0%)	9 (26.5%)	
180°	17 (100%)	25 (73.5%)	0.021 ^4^
Olecranon osteotomy performed	14 (82.3%)	29 (85.3%)	0.785 ^1^
Type of fixation of the osteotomy			
Tension band	12 (85.7%)	20 (69.0%)	
Cannulated screw	2 (14.3%)	9 (31.0%)	0.213 ^4^
Management of ulnar nerve			
Transposition	7 (41.2%)	5 (14.7%)	
In situ release	10 (58.8%)	29 (85.3%)	0.042 ^4^
MEPS	82.5	81.1	0.776 ^5^
Range of motion	99.7 degrees	101.0 degrees	0.887 ^2^

*p*-values were obtained using the chi-square test (^1^), the likelihood ratio test (^3^), Fisher’s exact test (^4^), Student’s *t*-test (^2^), and the Mann–Whitney U test (^5^) for continuous variables. AO/OTA, AO/OTA fracture classification system; MEPS, Mayo Elbow Performance Score.

**Table 3:** Studies evaluating ulnar neuropathy following open reduction and internal fixation of distal humerus fractures.

Study, Year	Mean Age (Y)	Sex	Follow-Up (M)	Secondary Surgical Procedure	In Situ	Transposed	Postoperative Ulnar Neuropathy	*p*-Value
This study	56	26 m	13	17 (33%)	39	12	17 (33%)	0.042
		25 w						
Vazquez et al., 2010 [28]	52	27 m	21	25 (36%)	22	47	11 (16%)	>0.05
		42 w						
Worden and Ilyas, 2012 [30]	46	15 m	13	NA	12	12	9 (37%)	>0.05
		9 w						
Chen et al., 2010 [23]	46	75 m	12	34 (25%)	89	48	24 (17%)	0.003
		62 w						
Wiggers et al., 2012 [34]	57	40 m	NA	42 (39%)	50	57	17 (16%)	0.01
		67 w						
Ahmed et al., 2020 [37]	36	73 m	11	NA	69	28	20 (21%)	0.027
		24 w						
Dehghan et al., 2021 [35]	53	23 m	12	11 (19%)	31	27	28 (48%)	>0.05
		35 w						
Oshika et al., 2023 [36]	56	39 m	11	NA	58	58	34 (29%)	>0.05
		77 w						

NA, not available; Y, years; M, months; m, men; w, women.

## Data Availability

The original contributions presented in this study are included in the article. Further inquiries can be directed to the corresponding author.

## Abbreviations

The following abbreviations are used in this manuscript:

AO	Arbeitsgemeinschaft für Osteosynthesefragen
OTA	Orthopedic Trauma Association
ORIF	Open reduction and internal fixation
ROM	Range of motion
MEPS	Mayo Elbow Performance Score
DHF	Distal humerus fracture
SD	Standard deviation
